# Integrating deep learning and field validation into a decision support system for Northern Corn Leaf Blight management in maize

**DOI:** 10.1186/s12870-026-08967-z

**Published:** 2026-05-19

**Authors:** Jadesha G., Anurag Dhole, Deepak D., Vishwanath K., Lohithaswa H. C.

**Affiliations:** 1 Plant Pathologist, College of Agriculture, GKVK, UniversityofAgriculturalSciences, Bangalore, India; 2 Department of Mechatronics, Manipal Institute of Technology, Manipal Academy of Higher Education, Manipal, India; 3 Special Officer (Seeds), National Seed Project, GKVK, University of Agricultural Sciences, Bangalore, India; 4 Zonal Agricultural Research Station, GKVK, UniversityofAgriculturalSciences, Bangalore, India

**Keywords:** Maize disease, Precision agriculture, Deep-learning, Decision support system, Fungicide advisory

## Abstract

Northern Corn Leaf Blight (NCLB; also, Turcicum Leaf Blight, TLB), caused by *Exserohilum turcicum* (teleomorph: *Setosphaeria turcica*), is one of the most destructive foliar diseases of maize worldwide, often causing severe yield losses under favorable conditions. We developed a maize-specific, web-based Decision Support System (DSS) for real-time NCLB detection and management (https://maize-nclb.streamlit.app/), integrating advanced deep-learning for automated diagnosis and fungicide advisory. Among thirteen Machine-learning and deep-learning models evaluated for classification, the Visual Geometry Group 16-layer convolutional neural network (VGG16) outperformed all others, achieving 94.0% accuracy, with balanced precision, recall, and F1-score of 0.94, and an AUC-ROC of 0.93. Confusion matrix analysis revealed minimal misclassification, with only 12 errors out of 357 samples, confirming the model’s high reliability in distinguishing healthy and infected plants, while Grad-CAM visualizations consistently highlighted biologically meaningful lesion regions, supporting the model’s interpretability and alignment with plant pathological symptoms. Field validation of DSS-guided fungicide recommendations (Azoxystrobin 18.2% + Difenoconazole 11.4% w/w SC) demonstrated significant benefits, reducing disease incidence to 6.8% compared with 67.4% in controls, achieving 90% disease reduction, and enhancing grain yield by 35.4% (8.55 t/ha), with a favorable cost–benefit ratio of 1:2.49. Seasonal disease progression analysis further confirmed DSS effectiveness, with cumulative disease burden reduced by approximately 85% compared with untreated control. These results highlight the potential of integrating deep-learning with field—validated management strategies into a practical DSS, demonstrating its potential for precision disease management in maize.

**Supplementary Information:**

The online version contains supplementary material available at 10.1186/s12870-026-08967-z.

## Introduction

Globally, maize (*Zea mays L.*) is a significant cereal crop used for food, feed and a variety of industrial processes, including biofuel production [7, 14]. Maize’s global importance also makes it vulnerable to several biotic stresses, particularly foliar diseases. Northern Corn Leaf Blight (NCLB), also known as Turcicum Leaf Blight (TLB), incited by *Exserohilum turcicum* (teleomorph: *Setosphaeria turcica*), is one of the most destructive foliar diseases of maize. The disease is widespread across all major maize-producing regions, including India, where favorable conditions such as high humidity and moderate temperatures facilitate its rapid spread. NCLB infection decreases the effective leaf area and photosynthetic performance, resulting in decreased grain filling and a loss of yield up to 22.5% worldwide [30]. Climate change is expected to increase the risk of Northern Corn Leaf Blight (NCLB) because warmer temperatures, higher humidity and altered rainfall patterns favor the fungus *Exserohilum turcicum*. Regions such as Asia and North America are particularly vulnerable due to their large maize production areas and conducive climates [24]. Among these, India confronts double challenges from water stress and shifting climate patterns that exacerbate the vulnerability of maize crops to NCLBs [6, 19]. These facts highlight the need for early and accurate detection of NCLB to safeguard productivity [14].

Conventional NCLB diagnostic tools viz., visual scouting of the field, ELISA and DNA based methods are time-consuming, laborious as well as costly to perform [16, 33]. What’s more they are heavily dependent on human expertise, that also bring in the factor of subjectivity and time [14]. Furthermore, the overlapping diseases with other foliage reduces accuracy under field conditions [4]. These constraints render such methods unsuitable for large-scale real-time surveillance in general farming systems.

Advances in artificial intelligence (AI), Machine-learning (ML) and Deep-learning (DL) for NCLB Detection: Advances in AI, ML and DL can provide strong solutions for plant disease diagnosis. Convolutional Neural Networks (CNNs) have demonstrated great performance in extracting both spatial and textural properties of leaf images, with classification accuracies exceeding 95% for several crops [32]. In the realm of CNNs, VGG16 has proven to be particularly effective for detecting subtle signs and symptoms of diseases across wide agro-climatic conditions [5]. Transfer learning makes it possible to re-train pre-trained networks such as VGG16 for locally-specific datasets, yielding even better performance in the case of data shortages [17, 35]. Furthermore, regularization techniques such as dropout and batch normalization help prevent overfitting, thereby improving model generalization [20]. Taken together, these advances contribute to sustainable agriculture by allowing early identification and precision management of NCLB.

Although deep-learning models provide high accuracy, they are often criticized for being “black boxes.” Interpretability is crucial for adoption among plant pathologists and farmers. Gradient- weighted Class Activation Mapping (Grad-CAM) helps address this challenge by generating heatmaps that highlight symptomatic leaf regions responsible for classification [9, 10]. Such visual explanations enhance trust, aid biological interpretation and ensure practical usability of AI-based systems in disease management [11, 26].

Combining AI- powered detection with digital tools can provide an approach for real-time response. Field-tested models, linked with web- based applications enable farmers to upload images of leaves and as a result receive diagnostic advice and tailored management recommendations such as fungicide advice [15, 31]. Such instruments do not simply identify disease but offer a means of action, thus connecting research and on-field application [8, 25].

The novelty of this work does not lie in proposing a new deep-learning architecture, but in the development of an integrated and field-validated decision support framework for maize NCLB management. The key contributions include:

1. Systematic benchmarking of machine-learning and deep-learning models for NCLB detection under real-field conditions;
2. Integration of explainable AI using Grad-CAM to ensure biological interpretability and user trust;
3. Deployment of a web-based DSS delivering real-time disease diagnosis and fungicide advisories; and
4. Multi-location field validation demonstrating agronomic and economic benefits of DSS-guided fungicide application.

In this context, the present study was undertaken with the following objectives: i. AI-Based Disease Classification: To evaluate and compare the performance of ML and DL models for NCLB detection in maize, with emphasis on classification matrices, confusion matrices and learning curves. ii. Model Interpretability and Visualization: To enhance biological interpretability of the best-performing model using Grad-CAM heatmaps of diseased leaf regions. iii. Digital Advisory Development: To develop and deploy a farmer-friendly web application that integrates the effective model with field findings, providing real-time NCLB detection and actionable fungicide-based disease management advisories. iv. Avoidable Yield Loss Assessment through DSS-Based Farm Advisory: To quantify avoidable yield losses in maize using DSS-based fungicide advisories, thereby validating the economic and practical utility of the developed decision support system.

## Material and methods

**Fig. 1:**
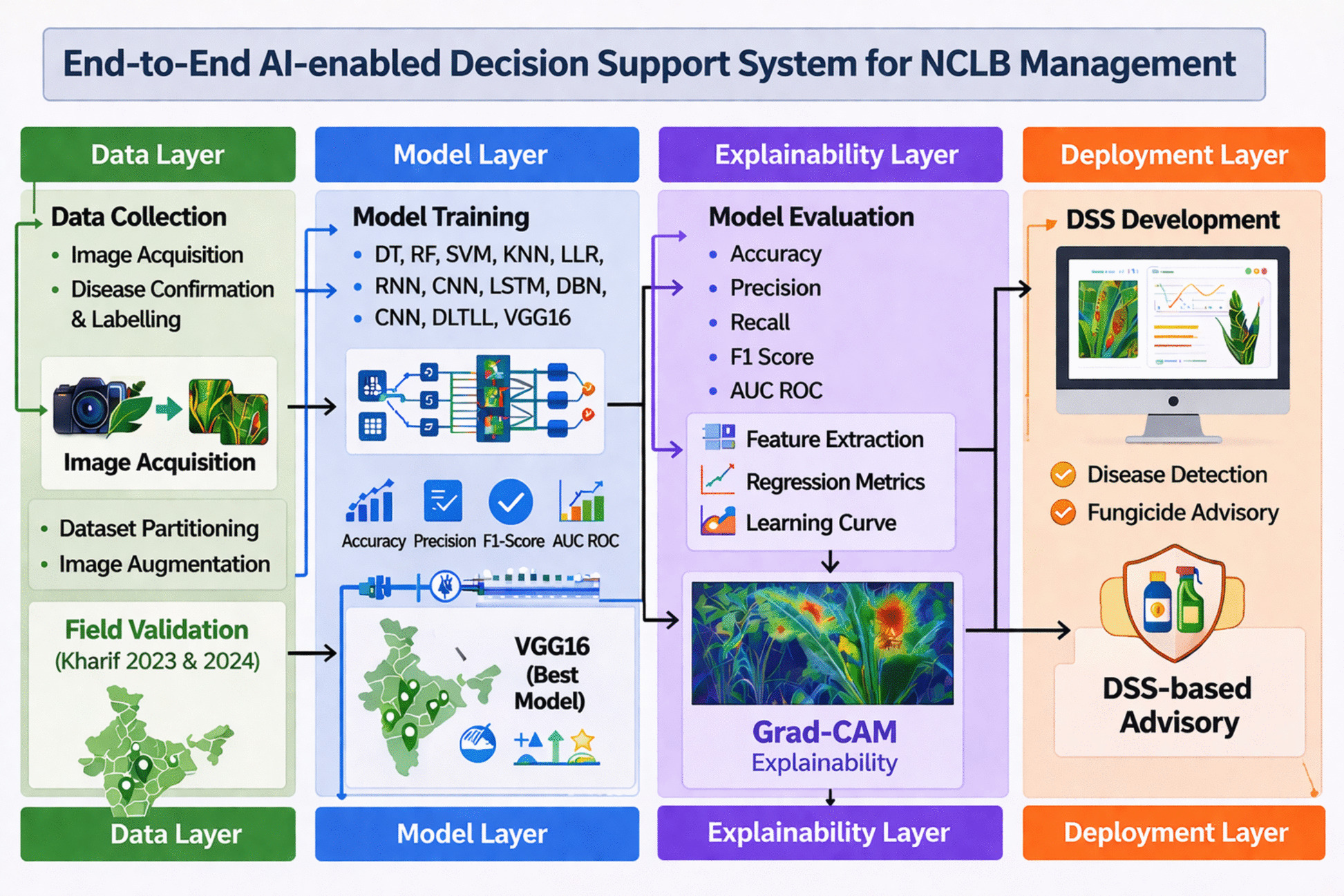
End-to-end framework of the AI-enabled decision support system for NCLB detection and management

The methodological framework employed in this study is illustrated in Fig. 1, depicting the systematic workflow for NCLB classification using ML and DL approaches.

The methodology encompasses sequential steps of A. Data acquisition and pre-processing, B. Model selection and Training, C. Performance evaluation, D. Decision Support System, E. Advisory Measures for NCLB Management. Each component is described in detail below.

### Dataset acquisition and pre-processing

#### Image acquisition

Maize leaf images representing both healthy and NCLB-infected specimens were systematically collected under diverse field conditions and controlled environments to ensure dataset representativeness and robustness. Field images were collected from commercial maize farms across multiple agro-climatic zones, capturing natural infections of Northern Corn Leaf Blight caused by *Exserohilum turcicum*, as well as from research farm plots where disease symptoms were generated through artificial inoculation following standard plant pathology protocols [12]. Images were collected during the Kharif (monsoon) seasons of 2023 and 2024 across multiple agro-climatic zones in Karnataka, India, including Mandya, Bangalore, Chikkaballapur, Hassan, and Mysuru districts. Details of sampling locations and agro-climatic characteristics are provided in Supplementary Table S1, and their geographic distribution is illustrated in Supplementary Figure S1. Images were captured using a high-resolution digital camera (Canon EOS R50 mirrorless camera with a 24.2 MP CMOS sensor and RF-S 18–45 mm lens) and smartphones equipped with ≥ 12 MP cameras, following standardized imaging protocols to ensure consistent image quality, appropriate focus, and minimal background interference. The dataset encompassed genetically diverse maize material, including inbred lines, MAGIC (Multi-parent Advanced Generation Inter-Cross) populations and commercial hybrids, captured across different crop growth stages (Supplementary Table S2). Images were acquired under varying environmental conditions, including changes in illumination, partial leaf occlusion due to canopy overlap, background clutter and natural field heterogeneity. The complete dataset comprised images taken under diverse lighting conditions and growth stages to enhance model generalizability. The final field dataset included a balanced representation of 3034 healthy and 3504 diseased samples to prevent class imbalance during model training. To further illustrate the diversity of the dataset under real-field conditions, representative sample images covering different disease stages and environmental variations are provided in Supplementary Table S3.

#### Disease confirmation and labelling

The field dataset was manually annotated by plant pathologists and certified disease specialists following proper diagnostic protocols for NCLB were followed based on standard guidelines described by Hooda et al. [12]. Microscopic examination was done to confirm the presence of *E. turcicum* conidia and characteristic symptoms such as elongated necrotic lesions with yellow halos. To ensure labelling accuracy, quality control procedures included double-blind validation by independent experts. Images with ambiguous symptoms or poor visual quality were removed from the dataset. The final dataset incorporated binary classification (healthy vs. diseased), enabling robust model training and evaluation.

#### Dataset partitioning

The field dataset was systematically partitioned using a 70:15:15 split ratio for training, testing and validation sets, respectively, ensuring balanced representation across all classes. Stratified sampling was employed to maintain consistent class distributions within each subset. The training dataset (70%) was utilized for model parameter learning, the validation dataset (15%) supported hyperparameter tuning and model selection, while the test set (15%) provided unbiased final performance evaluation.

#### Image pre-processing and augmentation

Image pre- processing methods were applied to normalize the field dataset and increase model robustness. All photos were resized to 224 × 224 pixels in order to maintain maximum computational efficiency and ensure compatibility with deep-learning systems. Pixel values were standardized to the range using min–max scaling to ensure dependable training convergence. To increase the size of the dataset and improve the model’s generalization skills, a variety of data augmentation methods were used, including random rotation (0–360° in 30° increments), flipping horizontally and vertically, brightness correction (± 20%) and contrast enhancement (0.8–1.2 ×). Data augmentation was applied exclusively to the training dataset to improve model generalization, while the validation and test datasets were subjected only to rescaling to ensure unbiased evaluation. The augmentation pipeline included rescaling of pixel values to the range [0,1], random rotations up to 20°, random zoom within a range of 0.2, and horizontal flipping. These transformations were applied in real time during training using the Keras ImageDataGenerator in a defined sequential order.

### Model selection and training

#### Algorithm selection and implementation

Thirteen distinct machine-learning and deep-learning algorithms were systematically evaluated for NCLB classification to identify optimal approaches. Traditional machine-learning methods Decision Trees, Random Forest, Support Vector Machines, K-Nearest Neighbors, Logistic Regression, Gradient Boosting Machines, and XGBoost. Advanced neural network architectures comprised Artificial Neural Networks with multiple hidden layers, Convolutional Neural Networks, Long Short- Term Memory networks, Deep Belief Networks and Siamese Networks along with Deep-learning approaches with transfer learning, including VGG16, were also evaluated.

#### VGG16 architecture implementation

The VGG16 model employed in this study was initialized using weights pretrained on the ImageNet dataset and subsequently fine-tuned for NCLB detection using the field-collected maize leaf image dataset. Because of the VGG16 architecture’s shown performance in plant disease classification tasks, it was chosen as the main deep-learning model. Using transfer learning approaches, the pre-trained VGG16 model—which was initially built on field datasets was optimized for NCLB detection. Three fully connected layers with dropout regularization (p = 0.5), five max-pooling layers with 2 × 2 windows and thirteen convolutional layers with 3 × 3 filters made up the architecture. Softmax activation was used in the final output layer to compute the probability distribution, whereas Rectified Linear Unit (ReLU) activation functions were used in hidden layers to induce non- linearity. Batch normalization between convolutional layers was used to increase stability and speed up training.

#### Software environment and implementation

Python 3.8.10 was employed to implement the experimental framework, together with specific libraries for ML and DL applications. PyTorch 1.9.0 was used for certain model implementations, whereas TensorFlow 2.6.0 and Keras 2.6.0 served as the main deep-learning framework. Scikit-learn 1.0.2 provided conventional Machine-learning methods and assessment measures, whereas OpenCV 4.5.3 managed image processing tasks. Numerical calculations and data processing were made easier with NumPy 1.21.0 and Pandas 1.3.3. Seaborn 0.11.2 and Matplotlib 3.4.3 were used for statistical charting and visualization.

#### Hyperparameter optimization

In addition to grid and random search strategies, hyperparameter selection for deep-learning models were guided by validation-driven empirical tuning. Key hyperparameters, including learning rate, batch size, number of epochs, optimizer type, and dropout rate, were initially selected based on prior literature on plant disease image classification and subsequently refined using validation set performance. The Adam optimizer was employed with adaptive learning rate adjustment to ensure stable convergence. The final model was trained with a learning rate of 0.001, batch size of 32, and for 10 epochs. A dropout rate of 0.5 was applied to reduce overfitting. The input image size was fixed at 224 × 224 pixels, and binary cross-entropy was used as the loss function. Early stopping criteria based on validation loss were applied to prevent overfitting and to improve model generalization. This systematic validation-based tuning ensured reproducible and robust model performance under real-field variability. A fixed random seed (42) was used for dataset partitioning and model initialization to ensure reproducibility across experiments.

### Performance evaluation

#### Comparison of classification metrics

Algorithm performance was assessed using classification-based metrics to ensure a robust and comprehensive assessment. For all thirteen algorithms, standard classification metrics were used, including Precision, Accuracy, F1-Score, Recall, AUC-ROC, Specificity and Negative Predictive Value (NPV). These metrics collectively provided insights into both overall predictive performance and class-wise discrimination, accounting for the trade-off between sensitivity and specificity. Overall predictive correctness is measured by accuracy, which is the percentage of correctly predicted disease occurrences compared to total predictions [18]. To minimize false positives and show the dependability of positive classifications [25] defined precision as the percentage of accurate positive predictions among all positive predictions. Recall (sensitivity) represents the proportion of true positives identified among all actual positive cases, indicating the model’s ability to detect disease occurrences [25]. The F1-Score, which is the harmonic mean of precision and recall and offers a single reliable index of classification performance, was used to balance these metrics [27]. Furthermore, AUC-ROC was employed to assess discriminative power across different thresholds, while NPV and Specificity (true negative rate) provided further information on the models’ capacity to accurately identify and omit healthy samples. In order to provide a thorough performance analysis, a confusion matrix was also created for every model, summarizing prediction results versus real data [18].

#### Comparison of feature extraction

Feature extraction was performed to assess the ability of different ML and DL algorithms to capture discriminative patterns from maize leaf images affected by NCLB. All input images were pre-processed through resizing and normalization to ensure consistency in scale and pixel distribution before being used for feature extraction. For DL models, particularly CNN, features were derived from the final convolutional or fully connected layers, while traditional ML algorithms utilized handcrafted feature representations generated from the pre-processed images. The same field dataset split was used for all models to ensure consistency in the evaluation of their feature extraction capabilities and to ensure fairness. The performance of the extracted features was quantitatively evaluated using classification accuracy, defined as the proportion of correctly classified samples to the total number of samples. Accuracy values range from 0 to 1, where higher values indicate better discriminative ability of the extracted features. This approach enabled a systematic comparison of the representational strength of ML and DL techniques in distinguishing between healthy and diseased leaf samples. By incorporating feature extraction analysis into the methodology, the most reliable and physiologically relevant features were identified for subsequent classification tasks, thereby improving model selection and interpretability.

#### Learning curve analysis of VGG 16

To evaluate how algorithm performance varies with training set size, a learning- curve analysis was performed for the VGG16 classifier and the best-performing traditional Machine-learning models. The training data were incrementally sampled in 10% steps from 10 to 100% of the total training set, while the validation set remained fixed. For each subset, the models were trained using the established training pipeline, and the corresponding training and validation performance values produced during each run were collected automatically by the framework. These values were plotted against the proportion of training data using Matplotlib 3.4.3 to generate the learning-curve graphs.

#### Grad-CAM analysis of VGG 16

In this study, Gradient- weighted Class Activation Mapping (Grad-CAM) was utilized for interpretable visual explanations of the VGG16 model’s decision-making process. This approach produced class-discriminative localization maps that emphasized the most salient image regions contributing to classification decisions through analysis of gradient information from the top convolutional layer. Color-generated heatmaps were overlaid on maize leaf images as intensity maps, with high-intensity regions (red/blue) representing areas most strongly correlated with NCLB symptom presence and low-intensity regions indicating minimal diagnostic relevance. The visualization process ensured that heatmaps were generated at the same spatial resolution as the original images for accurate interpretation. In addition to qualitative visualization, a quantitative assessment was performed by estimating the proportion of highly activated Grad-CAM pixels overlapping with visibly diseased regions identified by plant pathology experts. This overlap- based measure served as a proxy to validate that model attention was consistently aligned with biologically meaningful symptom regions rather than background artifacts. These analyses contributed to biological validation by confirming that the model focused on disease-relevant features while minimizing attention to irrelevant background patterns, thereby supporting reliable and explainable diagnosis suitable for real- world deployment.

#### Decision support system

**Fig. 2:**
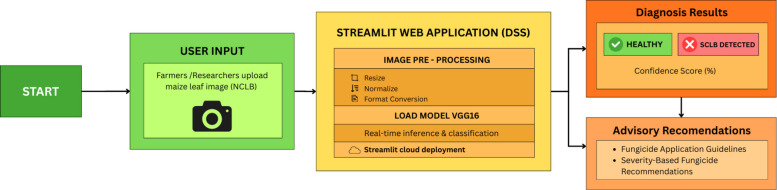
Decision support system architecture using VGG16 for NCLB detection

We developed a Decision Support System (DSS) based on the Streamlit framework due to its easy deployment, instantaneous response and user-friendly interface (Fig. 2).

The interactive system is developed for farmers, agricultural extension workers and researchers and available on the web with a user-friendly upload interface that allows uploading of the maize leaf images in order to ascertain disease status. When an image is uploaded, several preprocessing operations including resizing, normalization as well as format conversion are performed automatically to make it suitable for the fine- tuned VGG16 model pre-trained on NCLB datasets.

These operations ensure consistency and accuracy across a wide range of input images. Based on this, the processed images are then analyzed via the VGG16 model for real-time inference to classifying each sample as healthy or NCLB-infected. To increase its transparency, the system also outputs confidence scores for each prediction which represent its reliability. The results are presented in a simple, clean interface. In addition to diagnosis, the DSS offers actionable evidence-based recommendations related to fungicide application decisions and preventative agronomic practices, facilitating the timely management of NCLB in maize.

The deployed DSS corresponds to the final trained model (nclb_vgg_net16.keras) used in this study. To ensure reproducibility and long-term accessibility, the complete implementation, including model training, preprocessing, evaluation, and Grad-CAM visualization, along with deployment instructions, is publicly available at: https://github.com/anuragd02/NCLB-VGG16-Detection.

### Advisory measures for NCLB management

To evaluate the efficacy of advisory measures for managing NCLB disease, a field experiment was conducted during Kharif (monsoon season) 2023 and 2024. The study aimed to assess the impact of fungicide application on disease suppression and yield advantage. Two treatments were included: Azoxystrobin 18.2% + Difenoconazole 11.4% w/w SC and an untreated control (UTC). The experiment was conducted using an improved maize hybrid (HT-5402) under standard agronomic practices. The fungicide was applied at a concentration of 0.1% upon the initial appearance of disease symptoms. The study was carried out at research and farmers’ field locations in Karnataka, India, representing typical maize-growing agro-climatic conditions. Each treatment consisted of 12 replications, with each replication represented by a plot measuring 5 m in length with 6 rows, resulting in 12 plots per treatment. Observations on percent disease index (PDI) were recorded before spray, 15 days after spray (DAS), and 30 DAS, and percent reduction over control (PROC) was calculated to quantify the disease reduction. To evaluate cumulative disease development across the cropping season, disease progression was quantified using the Area Under the Disease Progress Curve (AUDPC), computed from PDI values recorded at successive observation intervals using the standard formula

$$AUDPC=\sum_{i=1}^{n-1}\left(\frac{{Y}_{i}+{Y}_{i+1}}{2}\right)\left({t}_{i+1}-{t}_{i}\right)$$

where Yi is the disease intensity at time ti. The calculation followed standard plant disease epidemiology methods [29]. Grain yield (t/ha) was recorded at harvest to determine the yield advantage and cost–benefit ratio of fungicide application. Statistical analyses using paired t-tests were performed to compare the fungicide treatment with the untreated control. Specifically, the paired t-test evaluated differences in PDI at each observation date, PROC, and grain yield between treated and untreated plots, providing statistical validation of the effectiveness of the advisory measures for NCLB management.

## Results

### Comparative ML and DL algorithms for NCLB detection

**Fig. 3:**
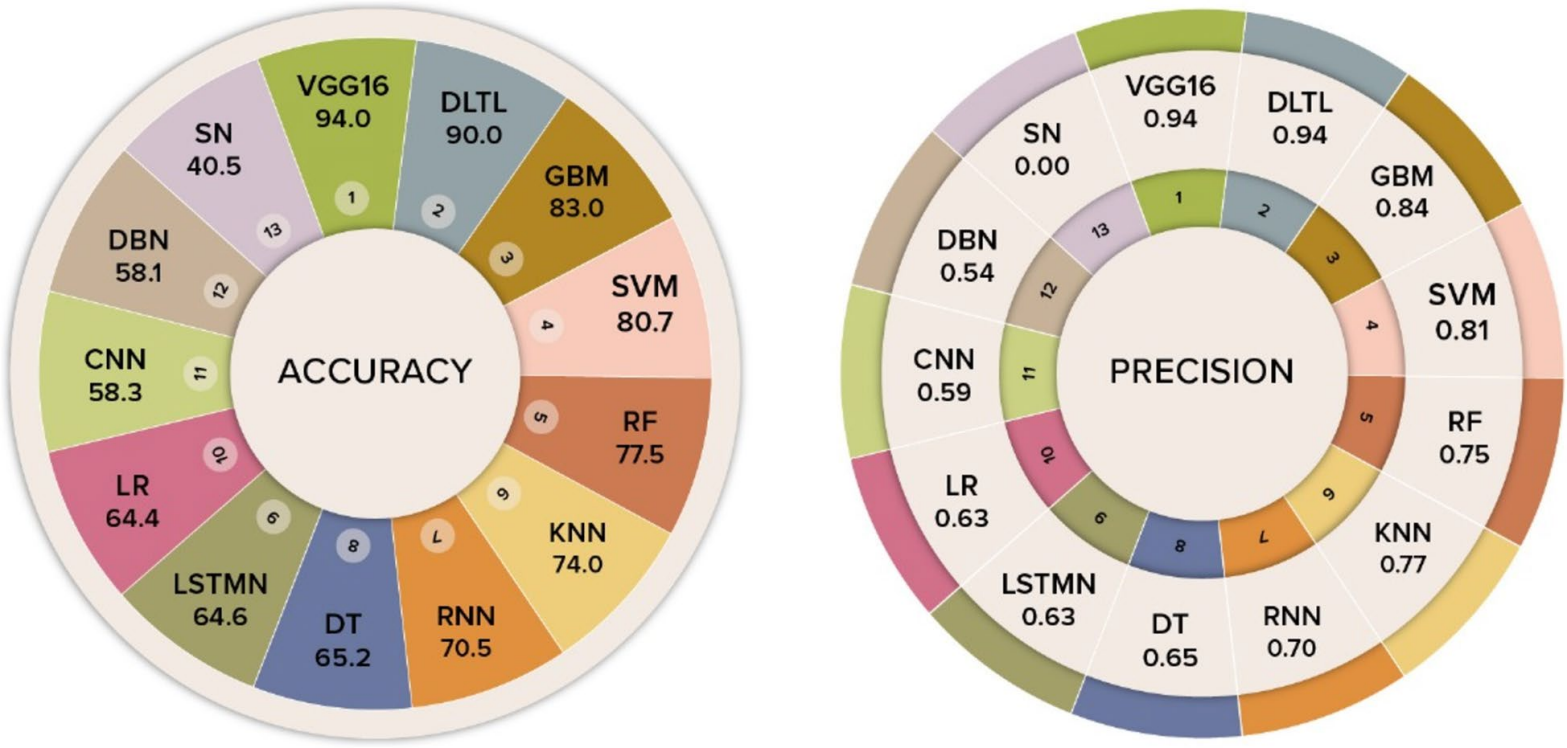
Accuracy and precision of algorithms in NCLB disease detection

**Fig. 4:**
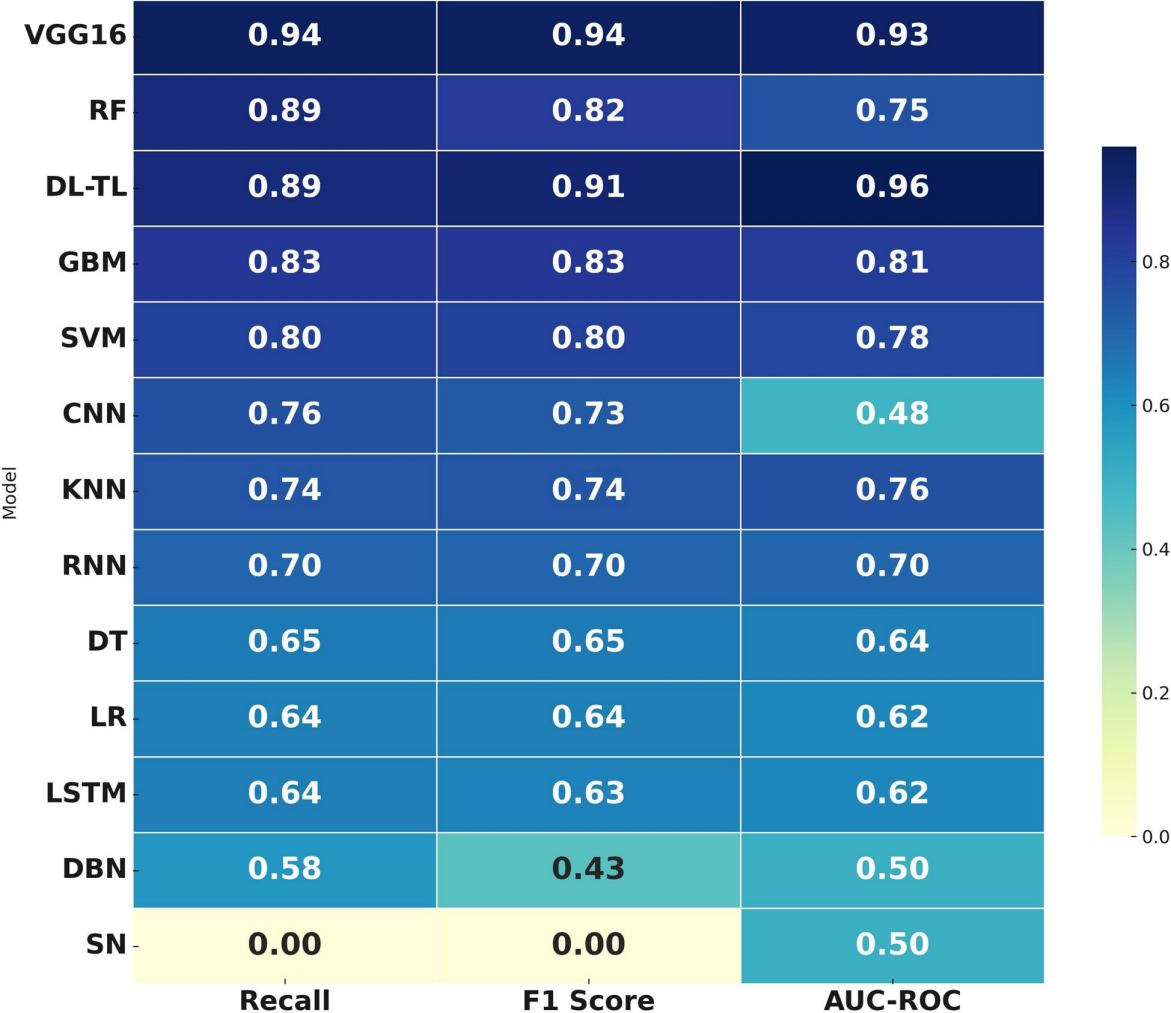
Recall, F1-score, and AUC–ROC of algorithms in NCLB disease detection

We evaluated 13 Machine-learning and deep-learning algorithms to detect Northern Corn Leaf Blight (NCLB) in maize, using accuracy, precision, recall, F1-score, and AUC- ROC as performance metrics. The results revealed substantial performance differences between models (Figs. 3 and 4). At the lower end, Siamese Networks performed very poorly, with only 40.5% accuracy and virtually no meaningful precision, recall, or F1-score, suggesting the model failed to classify the disease effectively. Deep Belief Networks (58.1%) and Convolutional Neural Networks (58.3%) also showed limited performance, reflected in low F1-scores (0.43 and 0.73) and weak AUC- ROC values (0.50 and 0.48). Among conventional Machine- learning models, Logistic Regression (64.4%), Long Short- Term Memory networks (64.6%), and Decision Trees (65.2%) achieved moderate accuracy, with reasonably balanced precision and recall (0.63–0.65). Performance improved with Recurrent Neural Networks (70.5%) and K-Nearest Neighbors (74.0%), the latter showing a precision of 0.77 and an AUC- ROC of 0.76.

Ensemble approaches performed even better. Random Forest reached 77.5% accuracy, with high recall (0.89) and an F1- score of 0.82. Support Vector Machines (80.7%) and Gradient Boosting Machines (83.0%) outperformed most earlier algorithms, maintaining balanced F1-scores (0.80–0.83) and improved AUC-ROC values (0.78–0.81).

Deep-learning models employing transfer learning produced the greatest performance improvements. This indicates that the model can reliably distinguish between healthy and diseased leaves under real-field conditions, making it suitable for practical disease diagnosis. The transfer learning model achieved 90.0% accuracy, along with strong precision (0.94), recall (0.89), F1-score (0.91), and the highest AUC-ROC (0.96), highlighting its superior ability to classify NCLB. Among all models tested, VGG16 performed the best, with 94.0% accuracy, precision, recall, and F1-score all at 0.94, and an AUC-ROC of 0.93. Overall, these findings demonstrate that deep-learning techniques, especially transfer learning and VGG16, offer extremely dependable and consistent performance, making VGG16 the best option for implementation in a real-time decision support system.

### Feature extraction performance of ML and DL models

**Fig. 5:**
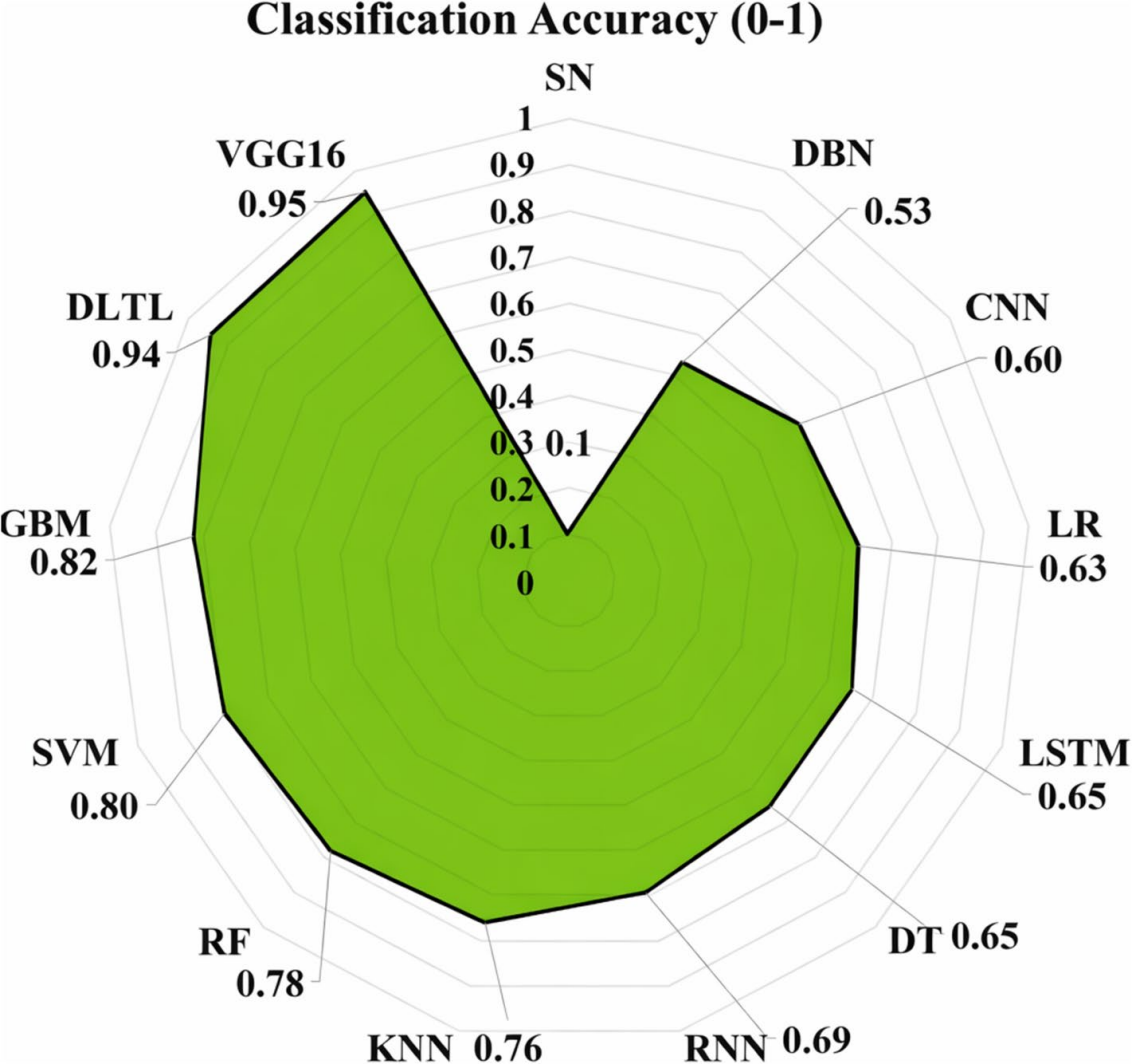
Comparative performance of ML and DL models in feature extraction, evaluated using classification accuracy

The results demonstrated a clear variation in feature extraction performance across models (Fig. 5). The performance values, expressed in terms of classification accuracy, ranged from 0.10 to 0.95. Among the models, Siamese Networks (0.10), Deep Belief Networks (0.53), and CNN (0.60) showed relatively lower accuracy, indicating limited effectiveness in capturing discriminative features. Traditional machine learning models such as Logistic Regression (0.63), LSTM (0.65), and Decision Trees (0.65) demonstrated moderate performance. K-Nearest Neighbors (0.76), Random Forest (0.78), and SVM (0.80) showed improved feature extraction capability, while Gradient Boosting Machines (0.82) achieved comparatively higher accuracy. In contrast, advanced deep learning approaches, particularly transfer learning (0.94) and VGG16 (0.95), achieved the highest classification accuracy, demonstrating their ability to capture complex and hierarchical disease-specific features from maize leaf images.

### Confusion matrix analysis of VGG16 for NCLB diagnosis

**Fig. 6:**
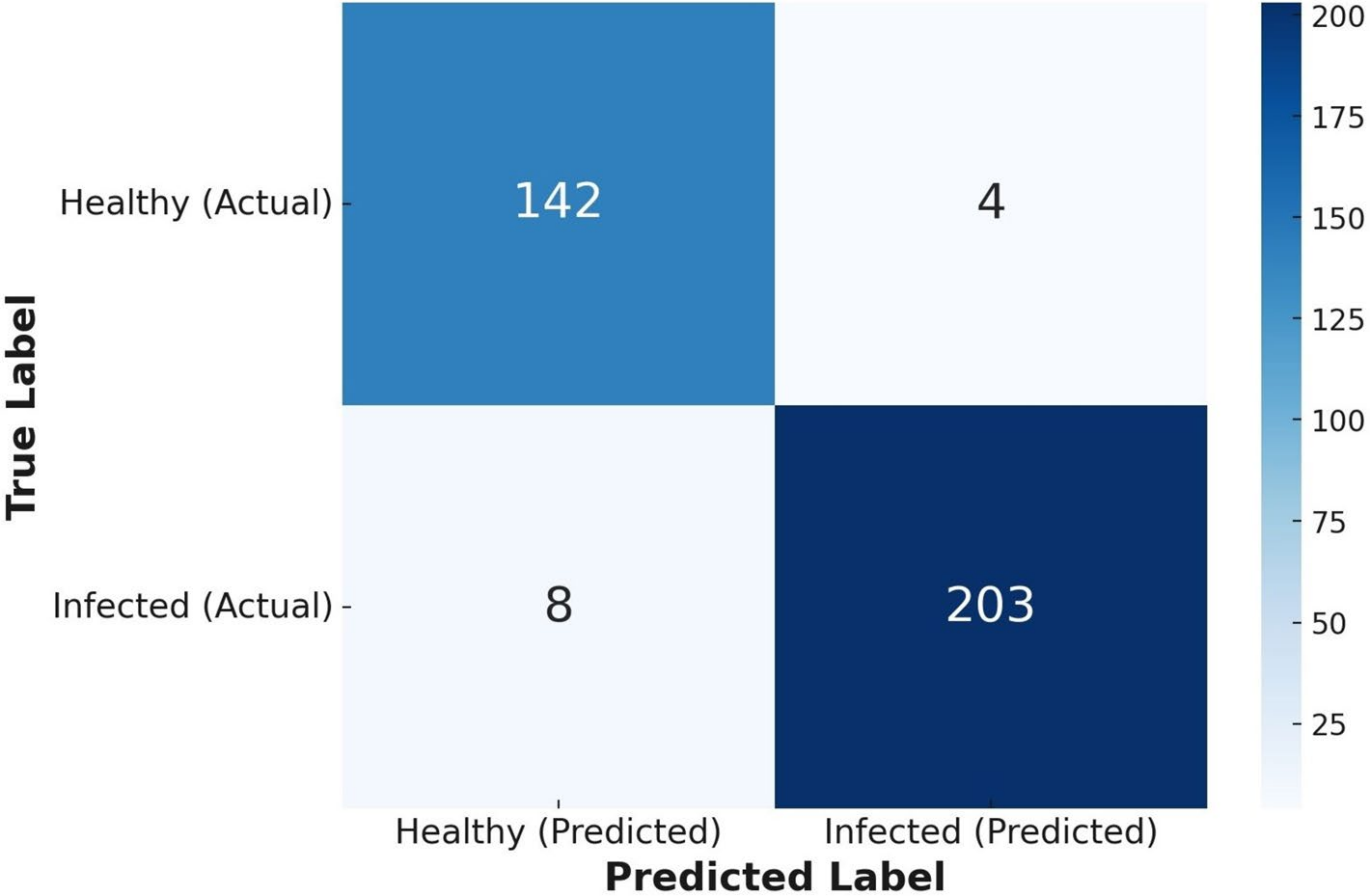
Confusion matrix analysis of VGG16 for NCLB detection

The confusion matrix offers detailed insights into the classification performance of VGG16 (Fig. 6). Out of the total test samples, the model correctly classified 142 healthy leaves as true negatives and 203 NCLB-infected leaves as true positives. Misclassifications were minimal, with only 4 healthy leaves incorrectly labelled as diseased (false positives) and 8 diseased leaves misclassified as healthy (false negatives). The very small number of false positives indicates that VGG16 has a low tendency to overestimate disease incidence, thereby reducing the risk of unnecessary fungicide applications or management interventions. Similarly, the limited false negatives highlight the model’s reliability in not overlooking infected samples, which is critical for early detection and timely disease management. Overall, the confusion matrix confirms the high sensitivity (recall) of VGG16 in detecting NCLB-infected plants, along with strong specificity in identifying healthy ones. This balance between sensitivity and specificity underscores the robustness of VGG16 as a diagnostic tool, reinforcing its potential for integration into precision agriculture decision-support systems.

### Learning curve analysis for VGG16 model in maize NCLB classification

**Fig. 7:**
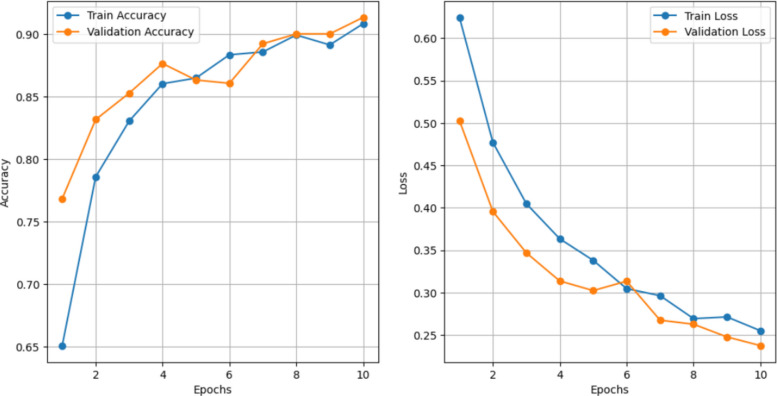
Learning curve patterns of VGG16 in NCLB detection

The learning curve analysis of the VGG16 model for NCLB classification demonstrates efficient model training and stable generalization. The training and validation accuracy curves indicate a consistent increase in performance over 10 epochs (Fig. 7). Training accuracy started at approximately 0.66 in the first epoch and steadily increased to 0.90 by the tenth epoch. Similarly, validation accuracy showed a corresponding rise, reaching 0.91, suggesting that the model generalized well to unseen data without substantial overfitting. The loss curves further support these observations. Training loss decreased from around 0.62 in the initial epoch to 0.25 by the tenth epoch, while validation loss followed a similar downward trend, declining from approximately 0.50 to 0.27. The parallel decrease in both training and validation loss, combined with the convergence of the accuracy curves, indicates effective learning and robustness of the VGG16 model for NCLB classification. Overall, the learning curve analysis confirms that the VGG16 model can capture discriminative features of NCLB infected and healthy maize leaves efficiently, achieving high accuracy and low error rates within a limited number of epochs. This highlights its suitability for practical applications in automated disease detection systems.

### Grad-CAM visualization of VGG16 for NCLB diagnosis

**Fig. 8:**
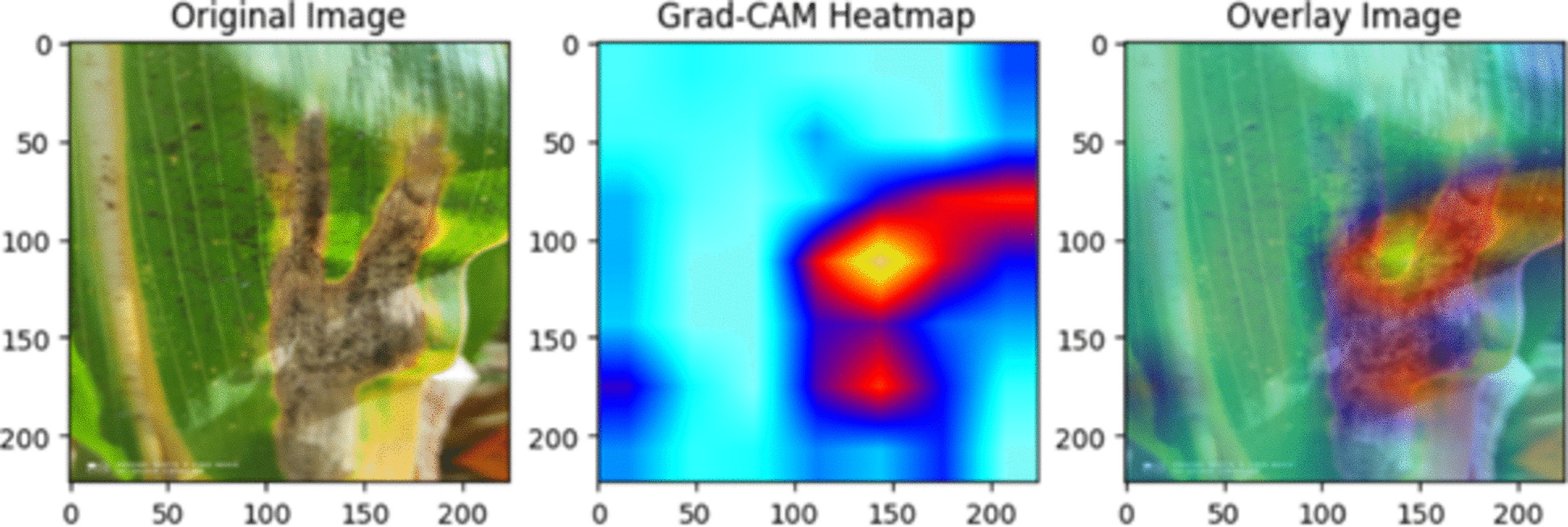
Grad-CAM–based interpretability of VGG16 in NCLB detection

To interpret the decision-making process of the VGG16 model, Gradient-weighted Class Activation Mapping (Grad-CAM) was applied to a representative maize diseases image (Fig. 8). The original image (left panel) shows clear necrotic lesions characteristic of NCLB. The Grad-CAM heatmap (middle panel) highlights areas of highest model attention in red, while regions of lower importance appear in blue. When overlaid on the original image (right panel), the heatmap demonstrates that VGG16 focuses precisely on the symptomatic lesion regions, effectively ignoring healthy tissue. This precise localization indicates that the model prioritizes biologically relevant disease features rather than background cues. The alignment between the lesion areas and model attention confirms the interpretability and reliability of VGG16’s predictions, supporting its application for automated and accurate NCLB detection in maize. This confirms that the model bases its predictions on visible disease symptoms, making the system more trustworthy and easier to interpret for non-expert users.

### Evaluation of advisory measures to minimize disease occurrence in NCLB

The fungicide advisory significantly reduced disease incidence compared to the untreated control (Table 1). PDI values were consistently lower in treated plots across all observation intervals. In Kharif 2023, the treated plots exhibited a PDI of 6.66% before spray, which slightly decreased to 5.00% at 15 DAS and 5.84% at 30 DAS. In contrast, the untreated control plots showed a significant increase in disease severity, with PDI rising from 5.55% before spray to 39.96% at 15 DAS and 64.31% at 30 DAS. The percent reduction over control (PROC) for the treated plots reached 90.9%, indicating a highly effective suppression of NCLB compared with the untreated control. Similarly, in Kharif 2024, the treated plots recorded a PDI of 6.66% before spray, 5.55% at 15 DAS, and 7.69% at 30 DAS, whereas the untreated control plots showed PDI values of 5.55%, 41.07%, and 70.48%, respectively. The corresponding PROC for the treated plots was 89.1%, confirming consistent disease control across two consecutive years. On average, across the two years, the treatment reduced the PDI to 6.8%, compared to 67.4% in the untreated control, resulting in an overall PROC of 90.0%. Paired t-test analysis confirmed that the reduction in disease severity under the fungicide-treated plots was statistically significant at both 15 DAS (p = 4.45 × 10^–6^; 95% CI: 33.96–35.97%) and 30 DAS (p = 5.75 × 10^–6^; 95% CI: 59.94—61.19%), demonstrating consistent and robust suppression of NCLB across seasons and replications. These results demonstrate that the Azoxystrobin + Difenoconazole treatment significantly suppressed NCLB and maintained low disease incidence compared to untreated plots. The high PROC values across both years highlight the effectiveness of the advisory measures in minimizing disease spread, thereby helping to minimize avoidable yield loss in maize.

**Table 1:** Reducing avoidable yield loss in maize through fungicide based advisory measures against Northern Corn Leaf Blight

Year	Treatments	PDI Before Spray (%)	PDI 15 DAS (%)	PDI 30 DAS (%)	PROC (%)	Yield (t/ha)	PIOC (%)	C:B Ratio
2023	T1: Untreated Control	5.55	39.96	64.31	0.00	6.05	–	1:1.64
	T2: Azoxystrobin 18.2% + Difenoconazole 11.4% SC	6.66	5.00	5.84	90.90	8.57	33.6	1:2.48
2024	T1: Untreated Control	5.55	41.07	70.48	0.00	5.93	–	1:1.52
	T2: Azoxystrobin 18.2% + Difenoconazole 11.4% SC	6.66	5.55	7.69	89.10	8.53	37.2	1:2.50
Mean	T1: Untreated Control	5.55	40.52	67.40	0.00	5.99	–	1:1.58
	T2: Azoxystrobin 18.2% + Difenoconazole 11.4% SC	6.80	5.28	6.77	90.00	8.55	35.4	1:2.49

*DAS* Days after spray, *PROC* Percent Reduction Over Control, *PIOC* Percent Increase Over Control, *C:B* Cost Benefit

Paired t-test analysis showed that reductions in percent disease index were statistically significant at 15 DAS (*p* = 4.45 × 10^–6^; 95% CI: 33.96–35.97%) and 30 DAS (*p* = 5.75 × 10^–6^; 95% CI: 59.94–61.19%) under DSS-guided fungicide application

### Seasonal disease suppression assessed using AUDPC

To evaluate cumulative disease development across the cropping season, disease progression was summarized using the Area Under the Disease Progress Curve (AUDPC). The untreated control plots recorded high seasonal disease pressure in both years, with AUDPC values exceeding 1100. In contrast, DSS-guided fungicide application drastically reduced cumulative disease development, with AUDPC values below 200 in both seasons (Table 2). On average, the fungicide advisory reduced seasonal disease burden by approximately 85% compared with untreated plots. These findings indicate that DSS-guided intervention effectively suppressed epidemic development throughout the season rather than merely delaying symptom expression.

**Table 2:** Seasonal disease pressure quantified using AUDPC

Year	Treatment	AUDPC*
2023	Untreated Control	1122.2
	Fungicide treatment	162.9
2024	Untreated Control	1214.5
	Fungicide treatment	194.4
Mean	Untreated Control	1168.3
	Fungicide treatment	178.7

^*^AUDPC calculated using disease severity observations at 0, 15 and 30 DAS

### Stability of DSS performance across seasons

To assess the consistency of DSS-generated recommendations under varying seasonal conditions, a two-way ANOVA was performed using year and treatment as factors. The analysis revealed a highly significant effect of treatment on seasonal disease pressure (p < 0.001), whereas the effect of year was not significant (Table 3).

**Table 3:** Two-way ANOVA for effects of year and treatment on seasonal disease pressure (AUDPC)

Source of variation	Degrees of freedom	F value	p-value
Year	1	2.14	0.19 ns
Treatment	1	865.72	< 0.001 ***
Year × Treatment	1	0.27	0.63 ns
Error	Remaining Degrees of freedom	–	–

*ns* non-significant; ^***^significant at *p* < 0.001. In the ANOVA model, “year” was treated as a categorical factor representing two independent cropping seasons (Kharif 2023 and Kharif 2024), rather than as a proxy for continuous environmental variables

Importantly, the Year × Treatment interaction was also non-significant, indicating that the fungicide advisory produced comparable levels of disease suppression in both seasons. This demonstrates that the DSS-generated recommendation was robust across seasonal variability and can be reliably applied under diverse field conditions.

### Relationship between disease suppression and yield gain

**Fig. 9:**
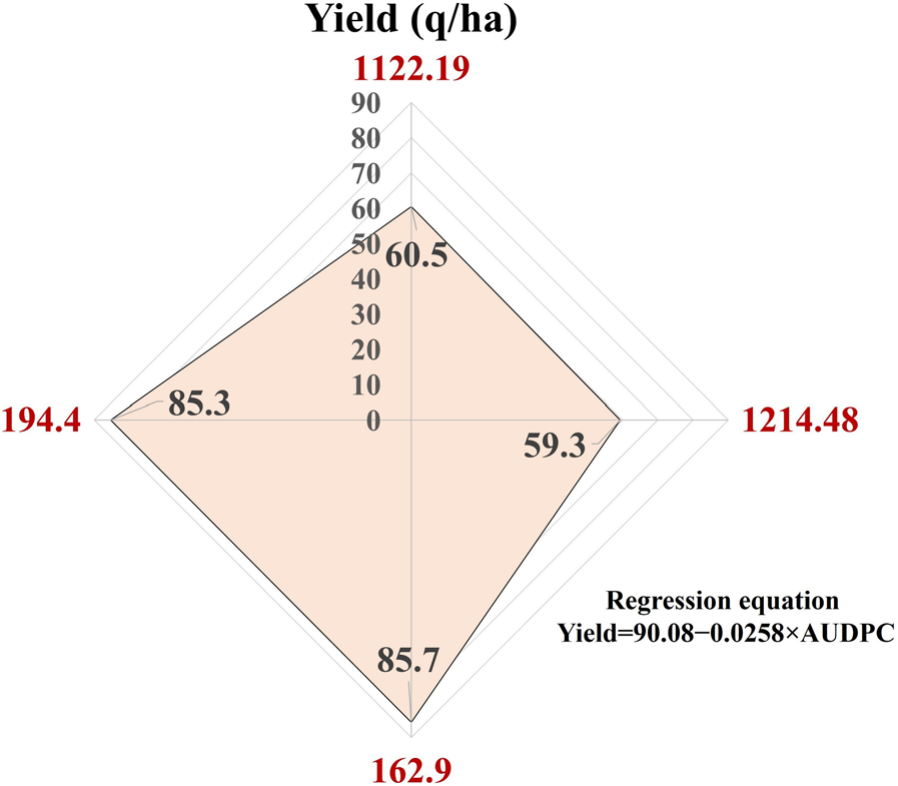
Relationship between cumulative disease pressure (AUDPC) and maize yield under DSS-guided management. Lower disease pressure is associated with higher grain yield

To determine whether reductions in disease burden translated into agronomic benefits, the relationship between cumulative disease pressure and grain yield was examined. Regression analysis revealed a strong inverse association between AUDPC and yield (Fig. 9), indicating that plots experiencing lower seasonal disease burden consistently produced higher yields.

The figure shows that lower disease pressure (AUDPC) is consistently associated with higher grain yield, indicating the effectiveness of timely disease management interventions. This confirms that DSS-guided fungicide application not only suppressed disease progression but also converted epidemiological advantages into measurable productivity gains. The close linkage between disease reduction and yield improvement highlights the practical value of the AI-enabled decision support system for minimizing avoidable yield loss in maize.

## Discussion

### Performance of machine-learning and deep-learning models in NCLB detection

**Fig. 10:**
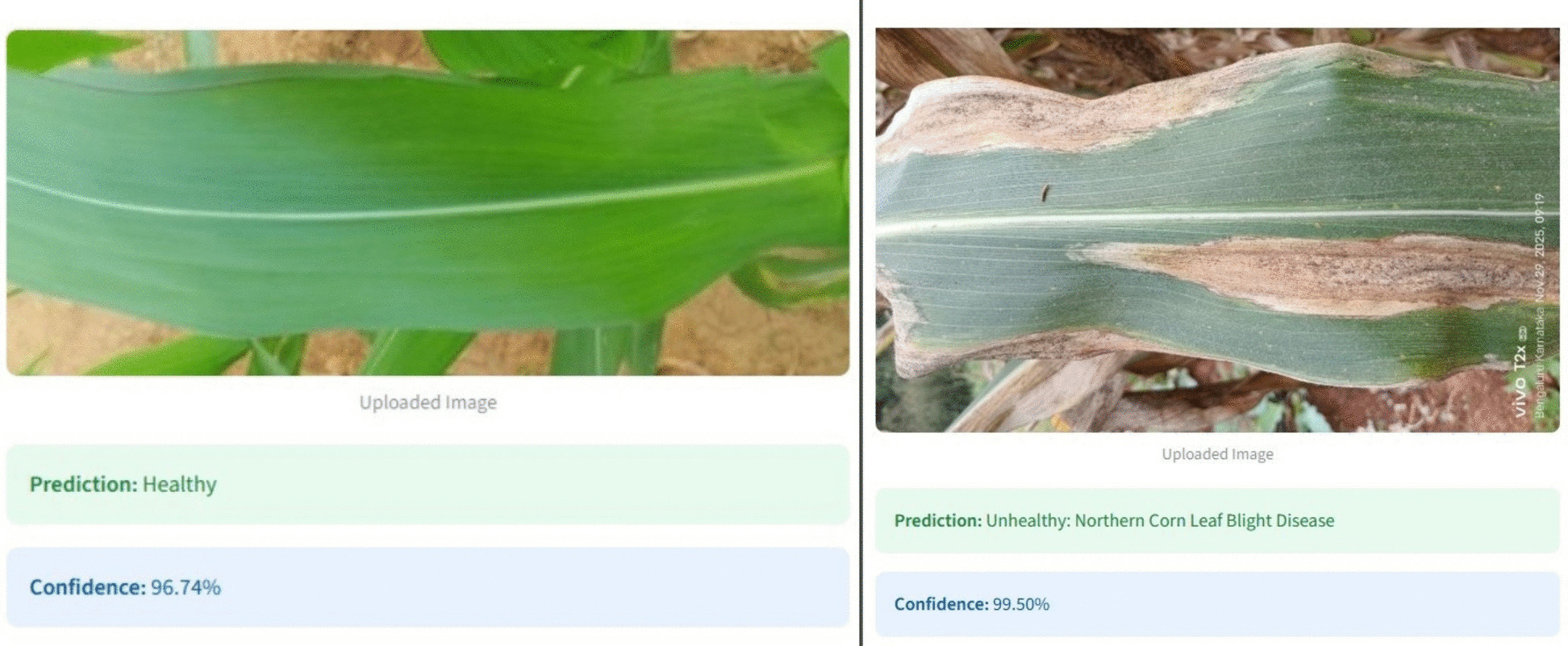
Representative outputs of the DSS showing classification of healthy and NCLB–infected maize leaves with prediction confidence

This study highlights a clear and consistent advantage of deep-learning (DL) models over traditional machine-learning (ML) approaches for the detection of Northern Corn Leaf Blight (NCLB) in maize under real- field conditions. Among 13 evaluated algorithms, the VGG16 convolutional neural network consistently achieved the highest classification accuracy (94%), balanced precision, recall, and F1-scores (0.94). Taken together, these metrics indicate that VGG16 performs well in categorical disease classification and demonstrates strong predictive consistency across heterogeneous field images (Fig. 10).

The superior performance of VGG16 and other DL approaches can be attributed to their inherent ability to automatically learn hierarchical and discriminative features directly from raw images. Unlike ML models such as decision trees and support vector machines, which rely on handcrafted feature selection, CNNs capture subtle spatial patterns, such as lesion morphology, chlorotic margins, and necrotic streaks, which are critical for accurate disease identification [14, 22, 23]. Handcrafted features, while effective under controlled or homogeneous imaging conditions, often fail to generalize when exposed to real-field variability such as illumination changes, leaf overlap, background clutter, and varying disease severity, thereby limiting their scalability in operational agricultural settings. Recent advances in DL architectures further reinforce their suitability for plant disease detection. Vision Transformers (ViTs), for example, leverage self-attention mechanisms to model complex spatial relationships within images, improving classification performance on heterogeneous and large-scale datasets [1]. Optimization strategies such as the Beluga Whale Optimization Mechanism have been integrated with CNNs to fine-tune network parameters, enhancing both accuracy and generalization [34]. Additionally, transfer learning approaches using architectures like ResNet50 enable rapid adaptation to new disease datasets while achieving accuracies exceeding 98% across multiple plant disease classes, demonstrating robustness and applicability to diverse crop–pathogen systems [3]. Collectively, these findings confirm that DL models sur- pass traditional ML techniques not only in predictive accuracy but also in robustness, scalability, and suitability for real-world agricultural disease surveillance. From a practical perspective, this means that farmers and field practitioners can rely on the system for accurate and timely disease detection without requiring technical expertise in artificial intelligence.

### Model interpretability and Grad-CAM visualization

While predictive accuracy is essential, interpretability is a critical requirement for the practical adoption of AI systems in agriculture, particularly when outputs directly influence disease management decisions. In this study, Grad-CAM visualizations demonstrated that VGG16 consistently focused on biologically relevant symptomatic regions of maize leaves, aligning with visible NCLB lesions and avoiding confounding background features. This strong correspondence between model attention and disease biology validates that the network is learning causally meaningful features rather than spurious correlations, thereby enhancing confidence in its predictions. Such biologically meaningful focus strengthens trust among farmers and agronomists, which is essential for the integration of AI tools into decision-support systems [10, 26]. Beyond this study, Grad-CAM and related visualization tools (e.g., Grad-CAM + +, Mask R-CNN) have been successfully applied in various plant disease detection contexts. For instance, Grad-CAM has been used to highlight critical lesion regions in leaf disease classification tasks, improving interpretability and diagnostic precision [28, 36]. Advanced deep-learning approaches combined with visualization techniques further enable precise localization of disease symptoms, supporting effective disease monitoring and management [13]. Thus, interpretability tools such as Grad-CAM act as an essential bridge between algorithmic predictions and observable plant pathology, ensuring that AI- based diagnostics remain transparent, explainable, and action- able for end users.

### Field validation and DSS-guided fungicide application

A key contribution of this study is the successful translation of AI-based disease detection into actionable field level disease management through a Decision Support System. The practical relevance of AI-based disease forecasting was validated through field trials integrating the VGG16 detection model into a DSS. Fungicide application based on DSS advisories—specifically, Azoxystrobin 18.2% + Difenoconazole 11.4% SC—led to a 90% reduction in NCLB incidence (PDI 6.8% versus 67.4% in untreated controls) and a corresponding 35.4% increase in grain yield (8.55 t/ha). The relatively higher grain yield observed in this study compared to the national average may be attributed to the use of an improved maize hybrid (HT-5402), along with optimal agronomic management practices and controlled experimental field conditions. cost–benefit ratio of 1:2.49 underscores the economic viability of DSS-guided interventions. These outcomes demonstrate that AI-driven disease forecasting, when integrated with decision support, can substantially outperform calendar-based or reactive fungicide application strategies. These findings are consistent with earlier studies reporting that forecasting-guided fungicide scheduling improves epidemic suppression, yield stability, and resource-use efficiency compared to conventional approaches [2, 6, 14, 21]. The broader applicability of DSS frameworks has also been demonstrated in other cropping systems. For example, in potato late blight management, DSS- driven fungicide strategies improved net returns while reducing unnecessary chemical applications [21]. Integrating the high- performing VGG16 model into a web-based DSS further enhances accessibility and scalability, providing farmers with real-time, field-specific advisories while minimizing labor, time, and chemical inputs [14, 15, 31]. When combined with IoT sensors, climate data, and remote sensing inputs, such DSS platforms have the potential to evolve into comprehensive crop health monitoring systems that support environmentally sustainable and precision-driven disease management.

### Practical implications, limitations and future perspectives

This study presents a holistic and operationally relevant framework that integrates deep-learning–based disease detection, explainable AI, and field-validated decision support into a single precision agriculture pipeline. Key strengths include:

- High-performing deep-learning backbone (VGG16) capable of reliable NCLB detection under real-field variability
- Interpretability via Grad-CAM, ensuring biologically meaningful and transparent predictions
- Deployment of a farmer-friendly web-based DSS delivering real-time fungicide advisories
- Field validation under natural conditions, capturing environmental and imaging variability
- Demonstrated agronomic and economic benefits, including 90% disease reduction and a favorable cost–benefit ratio (1:2.49)

These attributes collectively bridge the gap between laboratory-scale AI research and practical, field-deployable disease management solutions.

An additional limitation relates to the regional specificity of the dataset, which was primarily collected from Indian agro-climatic zones. While this may introduce geographic and environmental bias, transfer learning facilitates the extraction of generalized visual features; however, it may not fully account for variations across different agro-ecological regions without region-specific validation and model fine-tuning. Such representations may support adaptation of the proposed framework to new regions, maize-growing environments, and imaging contexts, but require further validation to ensure robustness and generalizability.

Despite these strengths, several limitations warrant consideration. Although the Streamlit-based DSS has been successfully deployed, the present study does not include a formal evaluation of user usability, farmer adoption, or end-user feedback. Since practical impact depends on user acceptance and ease of use, future research should incorporate structured usability assessments, farmer-centric validation trials, and participatory feedback studies to evaluate real-world effectiveness and adoption potential of the system.

Furthermore, the dataset used in this study was primarily collected from agro-climatic zones in India, which may limit the direct generalizability of the model to other maize-growing regions such as Sub-Saharan Africa or Latin America. Although transfer learning enables the extraction of generalized visual features, variations in environmental conditions, maize genotypes, and disease expression across regions may influence model performance. Therefore, further validation using multi-location and internationally diverse datasets is necessary to ensure global applicability and robustness. Additionally, the field validation was conducted over two consecutive seasons within a limited number of locations, which may not fully capture genotype × environment interactions and seasonal variability. Deep-learning models such as VGG16 require substantial computational resources for training and inference, which may limit adoption in resource-constrained regions without cloud or edge-computing support. Field implementation also depends on farmer awareness, adherence to recommended spray schedules, and availability of fungicides. Additionally, repeated application of the same fungicide combinations may increase the risk of resistance development, an aspect not evaluated in the present study.

Future research should focus on expanding datasets across diverse maize hybrids, growth stages, and agro-climatic zones to further enhance generalization. Developing multi-disease detection frameworks, lightweight deep-learning architectures for edge deployment, mobile-based interfaces, and integration with IoT-derived environmental data would improve accessibility and early warning capability. Long-term socio-economic and adoption studies are also essential to ensure sustained impact and scalability of AI-enabled DSS platforms.

## Conclusion

This study demonstrates the development and validation of an AI-enabled, web-based decision support system for real-time detection and management of Northern Corn Leaf Blight in maize. Among thirteen evaluated machine-learning and deep-learning algorithms, the VGG16 model achieved high classification accuracy and showed strong generalization under real-field conditions. The integration of Grad-CAM further strengthened the biological interpretability of the system by indicating that model predictions were based on symptom-relevant leaf regions rather than background artifacts.

Beyond algorithmic performance, the study highlights the practical value of integrating deep-learning diagnostics with field-validated management recommendations. DSS-guided fungicide application was associated with reduced disease incidence and improved grain yield while maintaining a favourable cost–benefit ratio, demonstrating the agronomic and economic relevance of the approach. The system also showed consistent suppression of seasonal disease development, contributing to a reduction in cumulative disease burden across seasons.

These findings suggest that AI-driven disease detection, when coupled with actionable advisory outputs, has the potential to bridge the gap between computational models and real-world crop management. Overall, the developed framework represents a promising and farmer-oriented approach for precision disease management in maize. However, further validation across diverse agro-climatic regions and cropping conditions is required to establish broader applicability. By combining automated diagnosis, explainable AI, and validated field interventions within an accessible web platform, this framework provides a robust foundation for future multi-disease monitoring systems and integrated digital agriculture tools aimed at improving crop health, productivity, and sustainability. Importantly, the system is designed to deliver simple, actionable outputs, ensuring that even non-expert users can effectively utilize the technology for disease management decisions.

DSS-guided fungicide application significantly reduced disease incidence and improved grain yield while maintaining a favourable cost–benefit ratio, demonstrating the agronomic and economic relevance of the approach. The system also consistently suppressed seasonal disease development, substantially reducing cumulative disease burden across seasons, thereby confirming its reliability under practical field conditions. These findings confirm that AI-driven disease detection, when coupled with actionable advisory outputs, can effectively bridge the gap between computational models and real-world crop management. Overall, the developed framework represents a scalable and farmer-oriented solution for precision disease management in maize. By combining automated diagnosis, explainable AI, and validated field interventions within an accessible web platform, this framework provides a robust foundation for future multi-disease monitoring systems and integrated digital agriculture tools aimed at improving crop health, productivity, and sustainability. Importantly, the system is designed to deliver simple, actionable outputs, ensuring that even non-expert users can effectively utilize the technology for disease management decisions.

## Supplementary Information

Supplementary Material 1.

Supplementary Material 2.

Supplementary Material 3.

Supplementary Material 4.

## Data Availability

The datasets generated and/or analyzed during the current study are not publicly available at this stage as they form part of an ongoing research project. However, the data are available from the corresponding author upon reasonable request for academic and non-commercial use. A permanent DOI-linked repository will be provided upon completion of the project.
