# Supplementary figures and images for: Integrating deep learning and field validation into a decision support system for Northern Corn Leaf Blight management in maize
Source: BMC Plant Biol. 2026 May 19;26:1179. doi: 10.1186/s12870-026-08967-z (PMC13352657; doi:10.1186/s12870-026-08967-z)

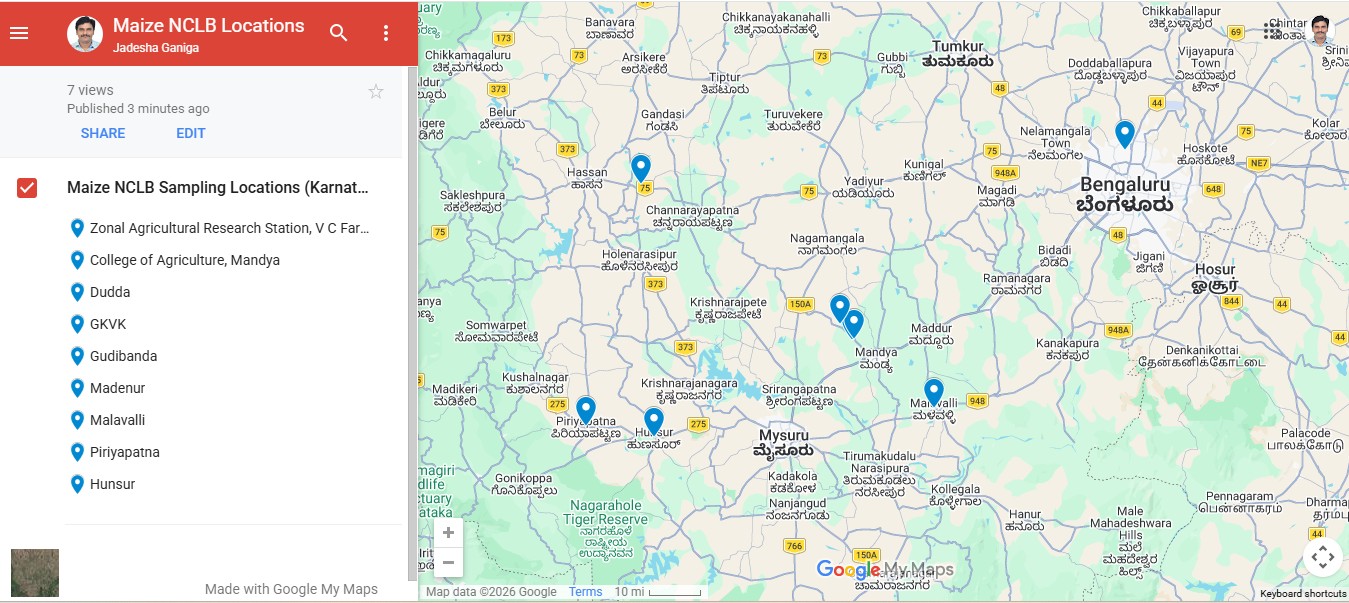

Supplement: Supplementary file 1 — Supplementary Material 1. [file 12870_2026_8967_MOESM1_ESM.jpg]
