# Supplementary material for: Integrating deep learning and field validation into a decision support system for Northern Corn Leaf Blight management in maize
Source: BMC Plant Biol. 2026 May 19;26:1179. doi: 10.1186/s12870-026-08967-z (PMC13352657; doi:10.1186/s12870-026-08967-z)
Supplement: Supplementary file 2 — Supplementary Material 2. [file 12870_2026_8967_MOESM2_ESM.docx]

**Supplementary Table S1. Sampling locations, coordinates, and agro-climatic characteristics of maize leaf image collection sites in Karnataka, India.**

| **Sl. No.** | **Location** | **Type of Site** | **District** | **State** | **Agro-climatic Zone** | **Approx. Coordinates** |
| --- | --- | --- | --- | --- | --- | --- |
| 1 | ZARS, V.C. Farm (C Block) | Research Farm | Mandya | Karnataka | Southern Dry Zone | 12.52°N, 76.90°E |
| 2 | College of Agriculture, V.C. Farm | Research Plot | Mandya | Karnataka | Southern Dry Zone | 12.52°N, 76.90°E |
| 3 | Dudda Village | Farmers’ Field | Mandya | Karnataka | Southern Dry Zone | 12.58°N, 76.92°E |
| 4 | GKVK, Bangalore | Research/Demonstration Field | Bangalore | Karnataka | Eastern Dry Zone | 13.08°N, 77.58°E |
| 5 | Gudibande Taluk | Farmers’ Field | Chikkaballapur | Karnataka | Eastern Dry Zone | 13.67°N, 77.70°E |
| 6 | ARS, Madenuru | Research Station | Hassan | Karnataka | Southern Transition Zone | 13.00°N, 76.10°E |
| 7 | Malavalli Taluk | Farmers’ Field | Mandya | Karnataka | Southern Dry Zone | 12.38°N, 77.08°E |
| 8 | Piriyapatna Taluk | Farmers’ Field | Mysuru | Karnataka | Southern Transition Zone | 12.33°N, 76.10°E |
| 9 | Hunsur Taluk | Farmers’ Field | Mysuru | Karnataka | Southern Transition Zone | 12.30°N, 76.30°E |
