# Supplementary material for: Integrating deep learning and field validation into a decision support system for Northern Corn Leaf Blight management in maize
Source: BMC Plant Biol. 2026 May 19;26:1179. doi: 10.1186/s12870-026-08967-z (PMC13352657; doi:10.1186/s12870-026-08967-z)
Supplement: Supplementary file 4 — Supplementary Material 4. [file 12870_2026_8967_MOESM4_ESM.docx]

Table S1. Representative sample images illustrating dataset diversity under real-field conditions

| **Category: Healthy (Normal leaf)** | |
| --- | --- |
| 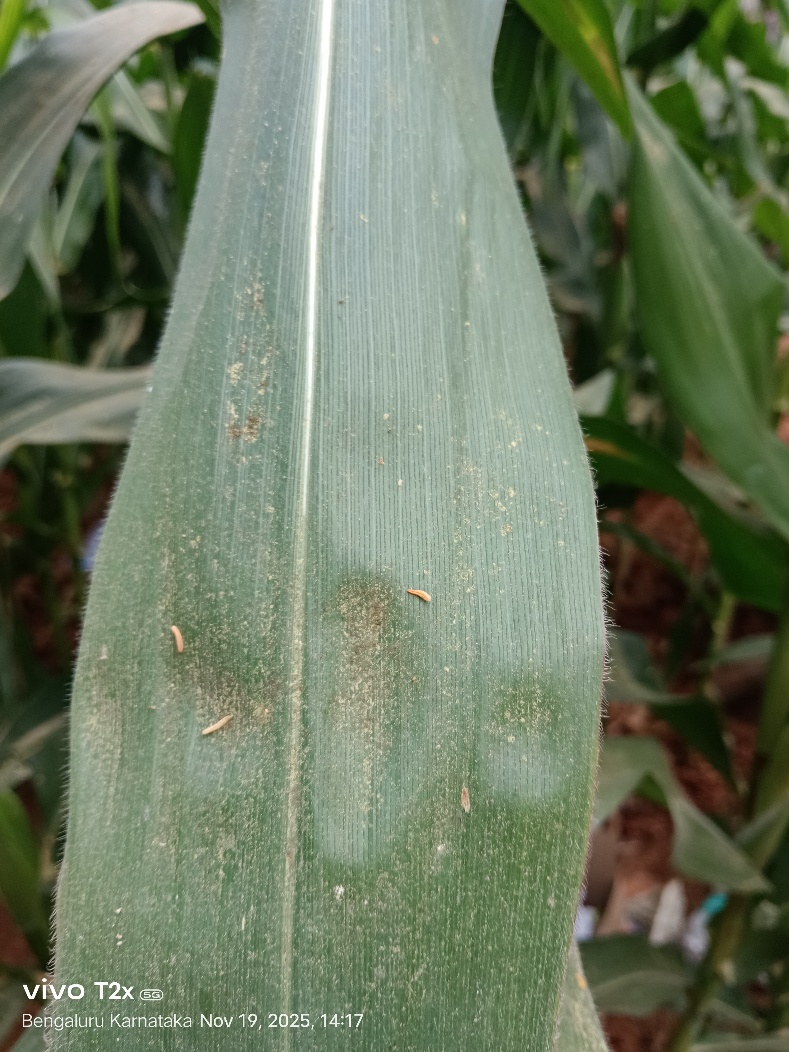 | 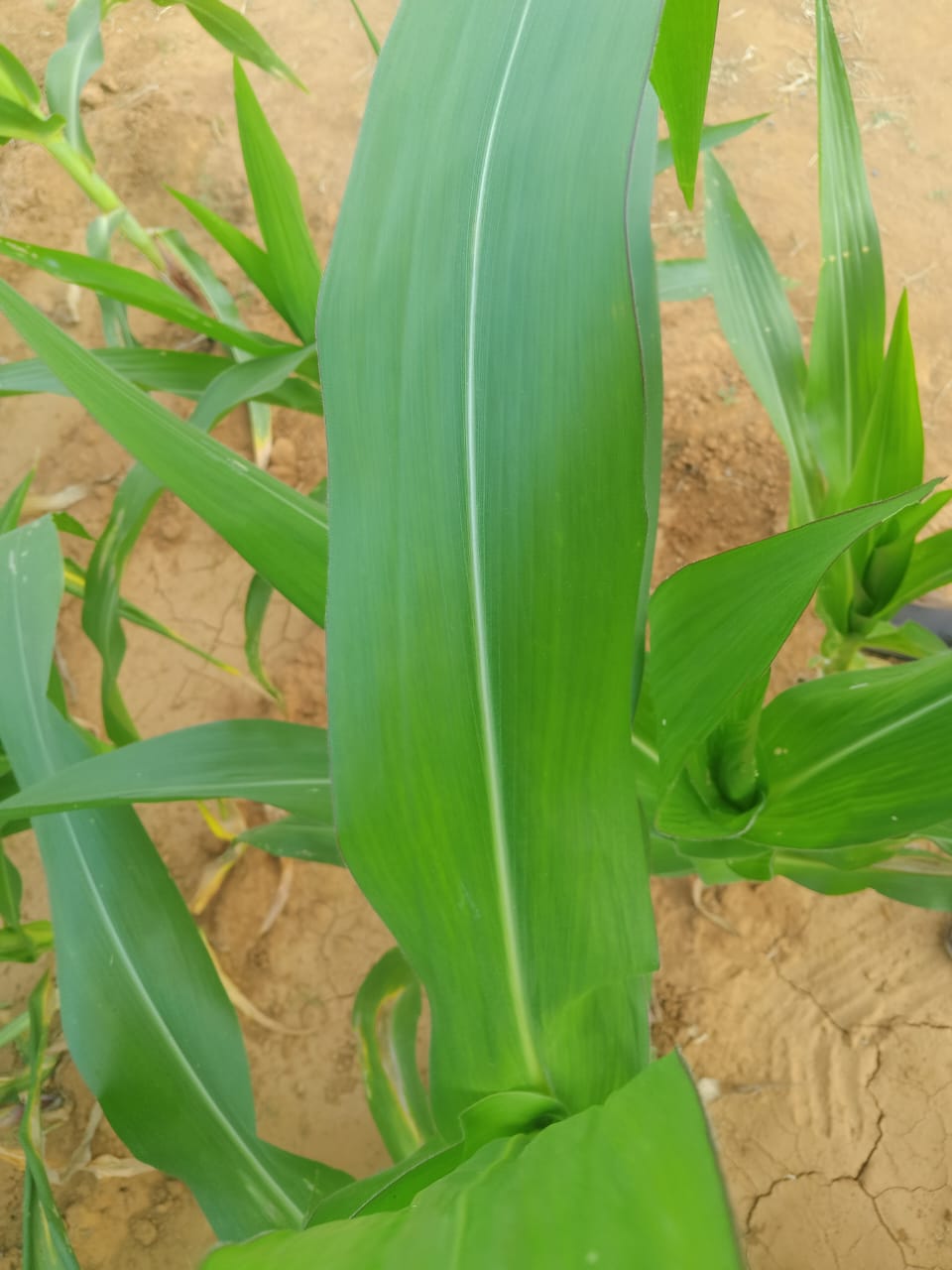 |

| **Category: Early NCLB (Mild lesion)** | |
| --- | --- |
| 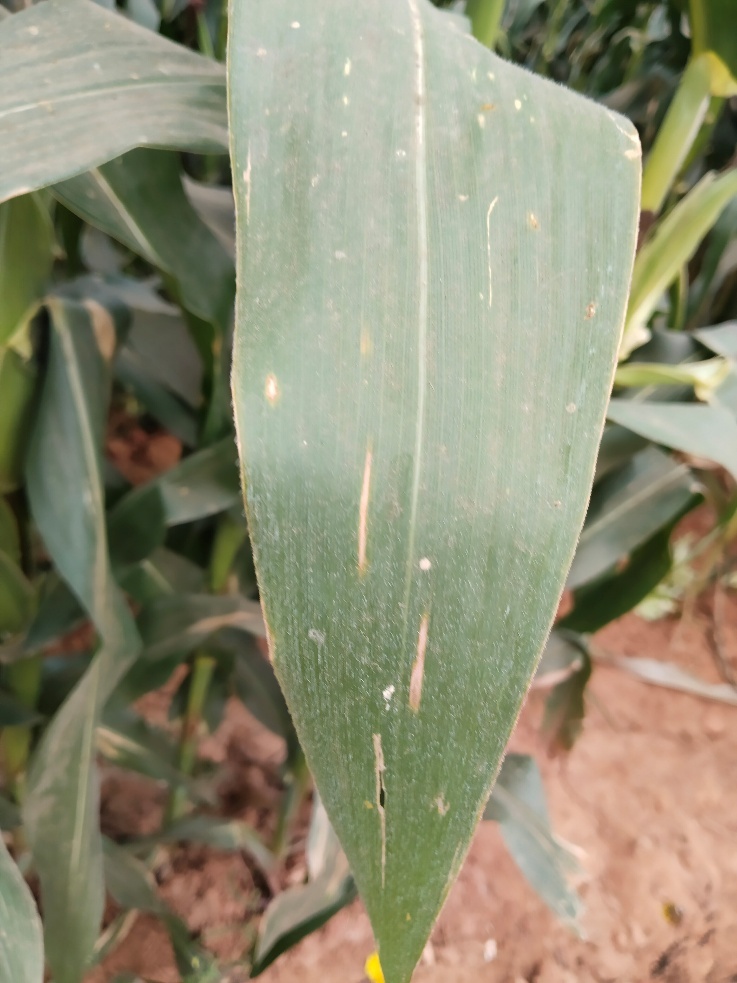 | 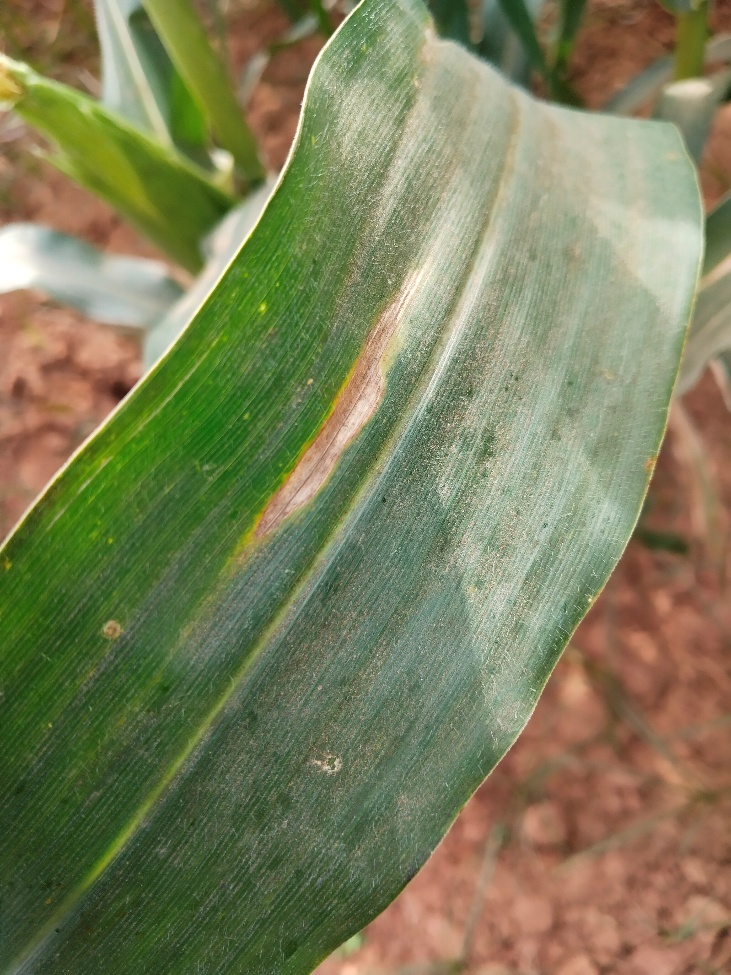 |

| **Category: Severe NCLB (Advanced necrosis)** | |
| --- | --- |
| 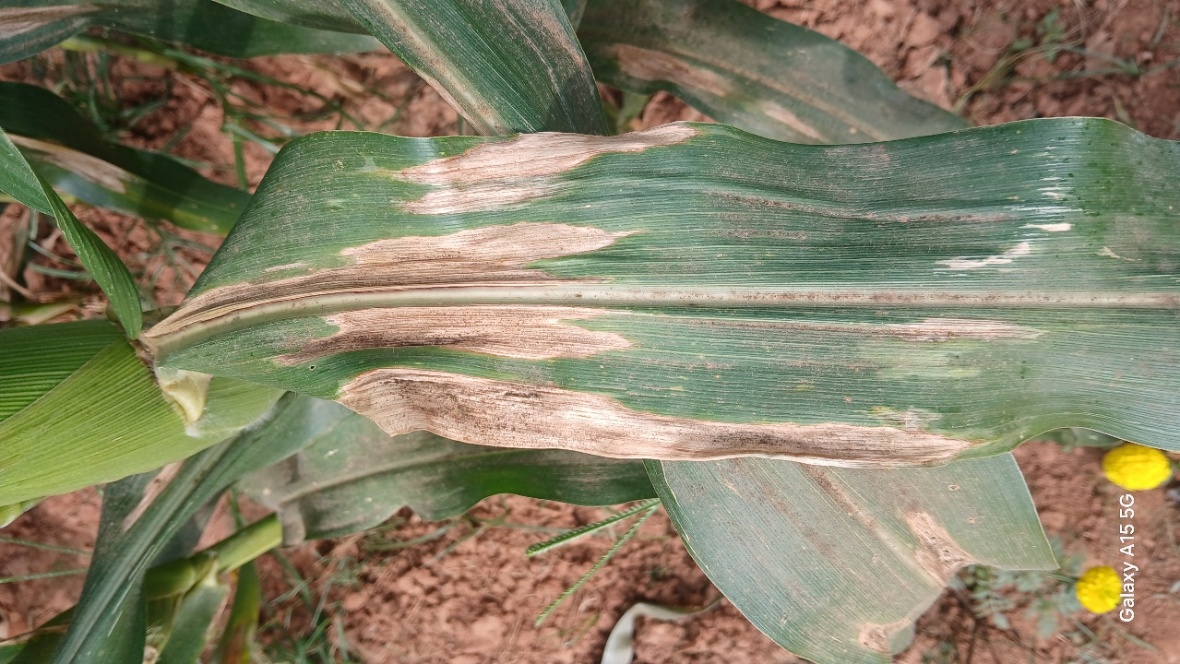 | 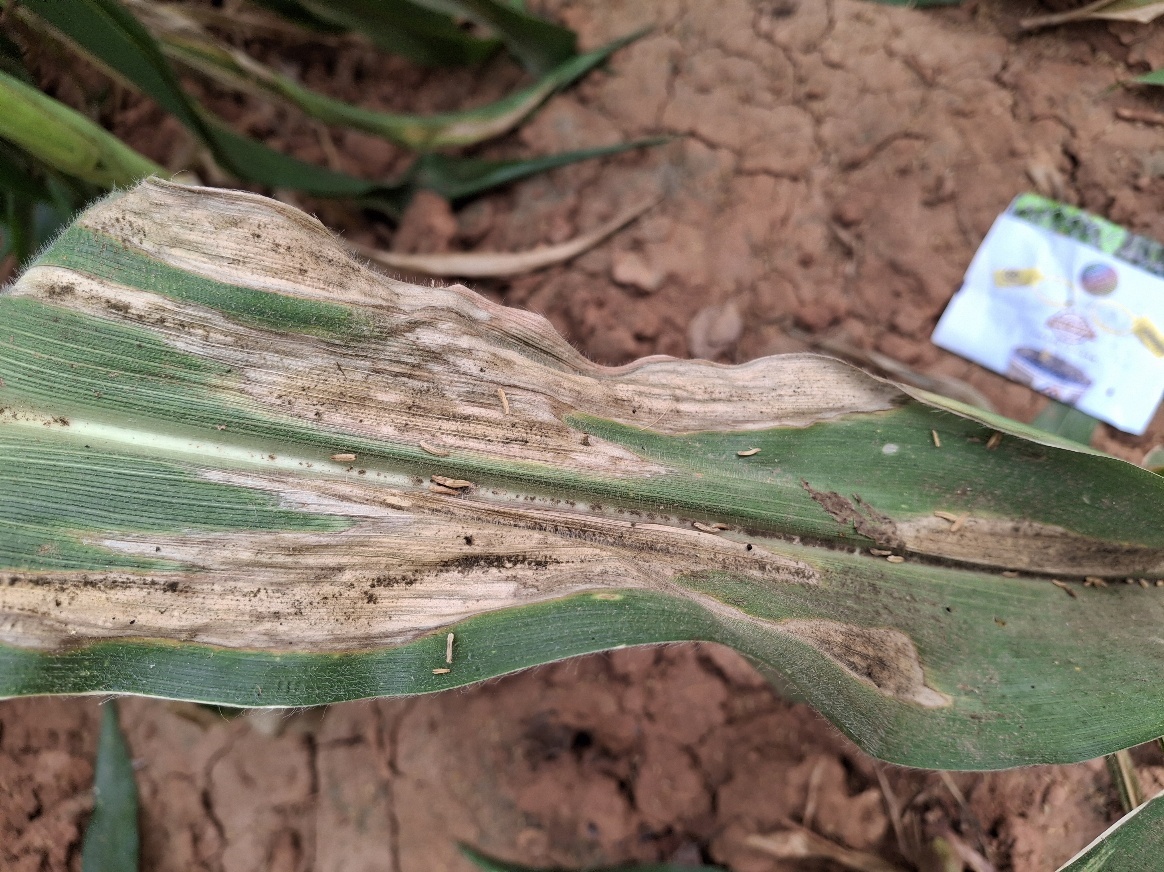 |

| **Occlusion: Severe NCLB (Leaf overlap)** | |
| --- | --- |
| 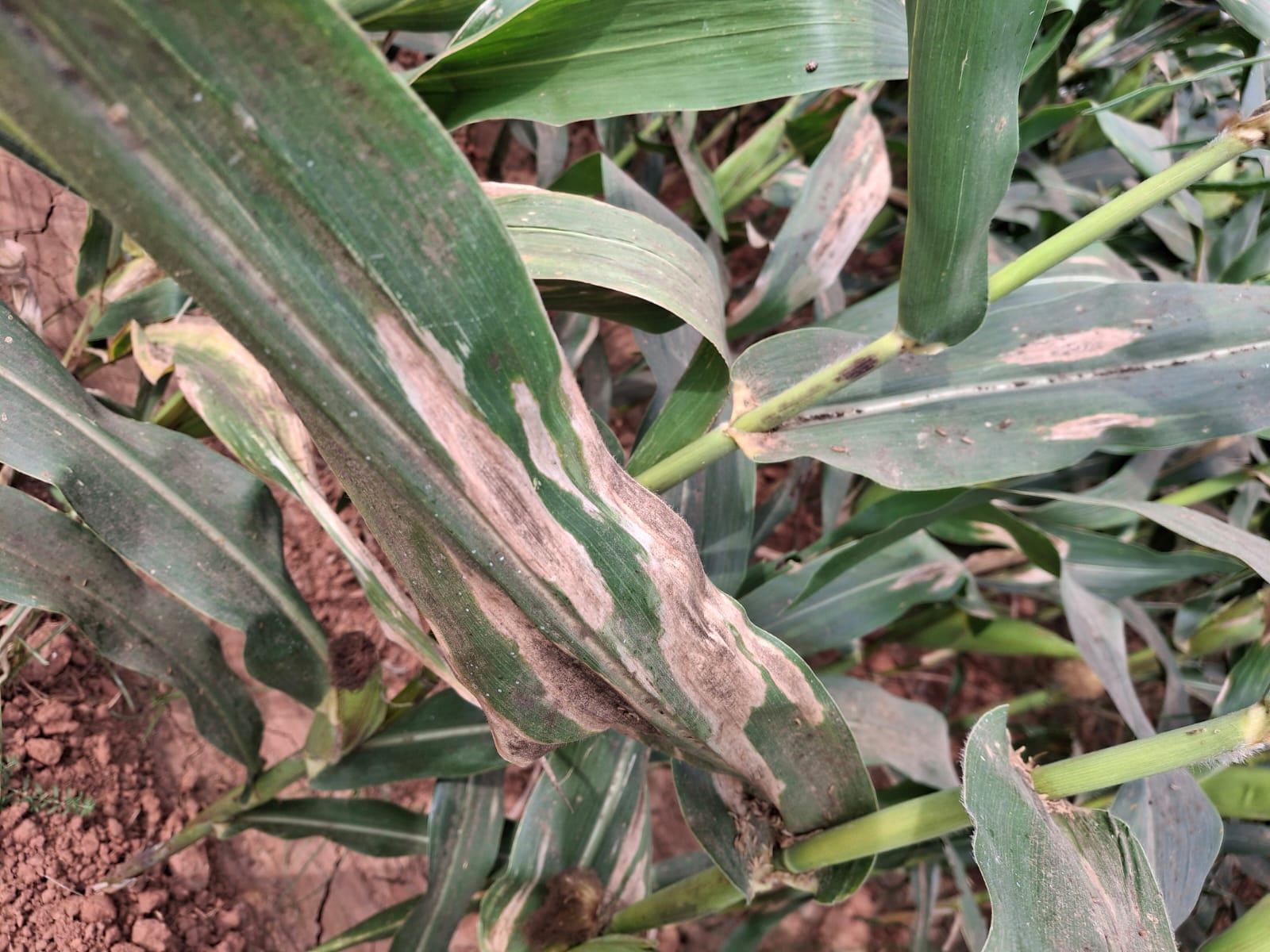 | 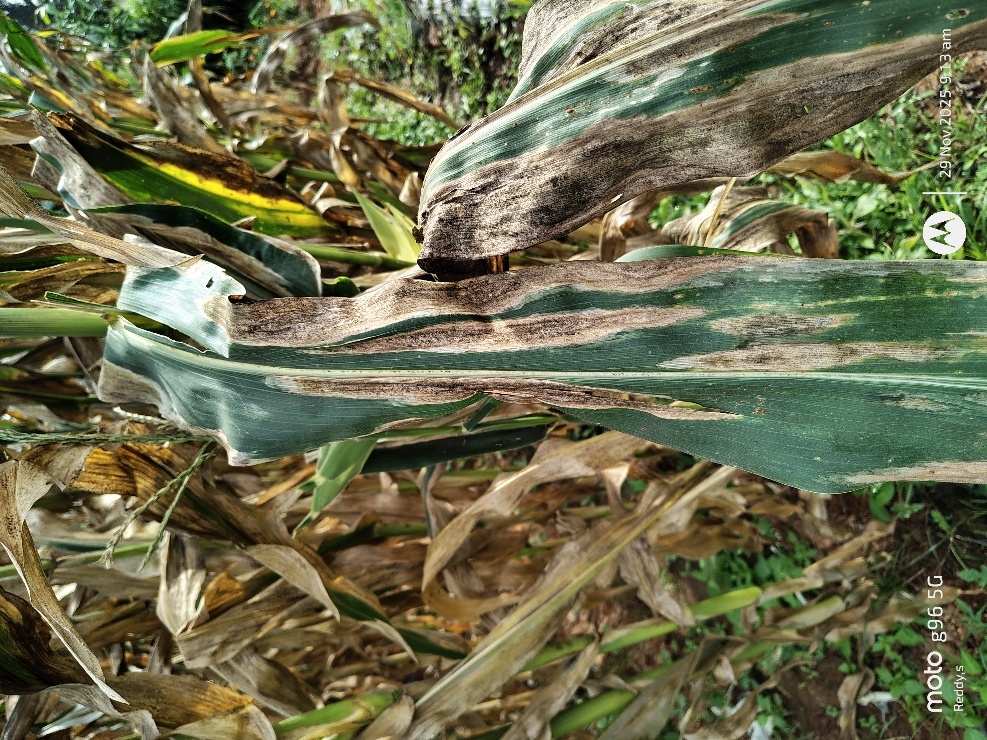 |

| **Illumination variation (Bright Sunlight)** | **Illumination variation (Shadow Sunlight)** |
| --- | --- |
| 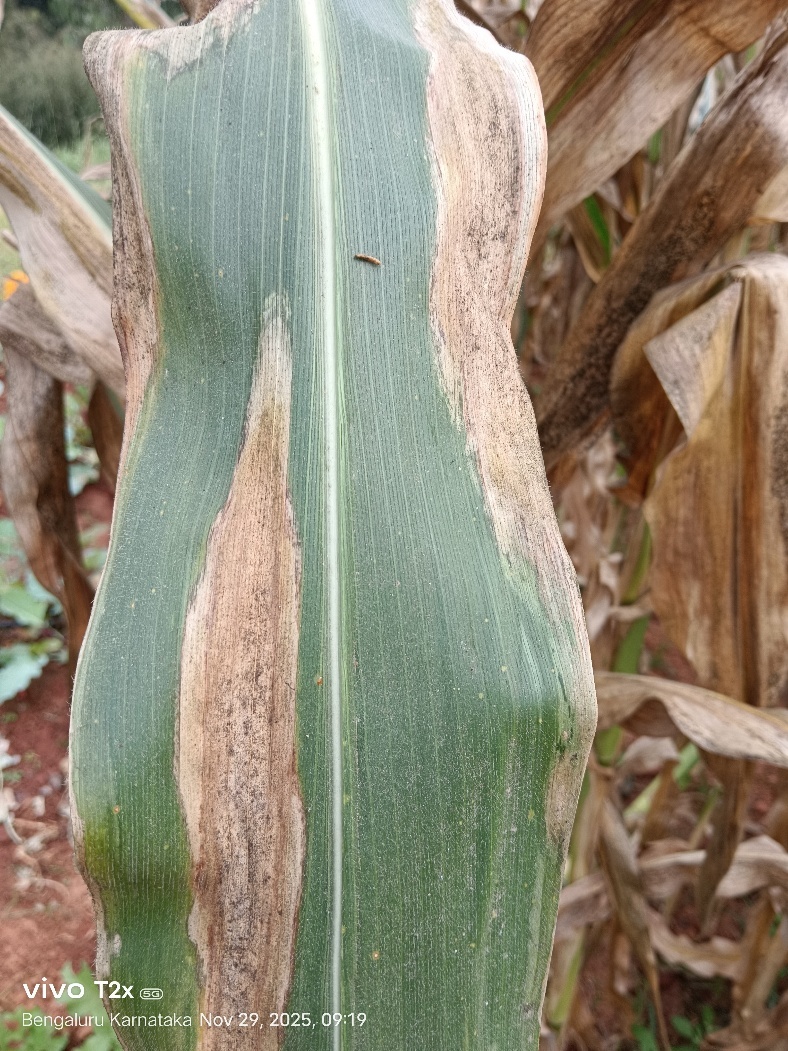 | 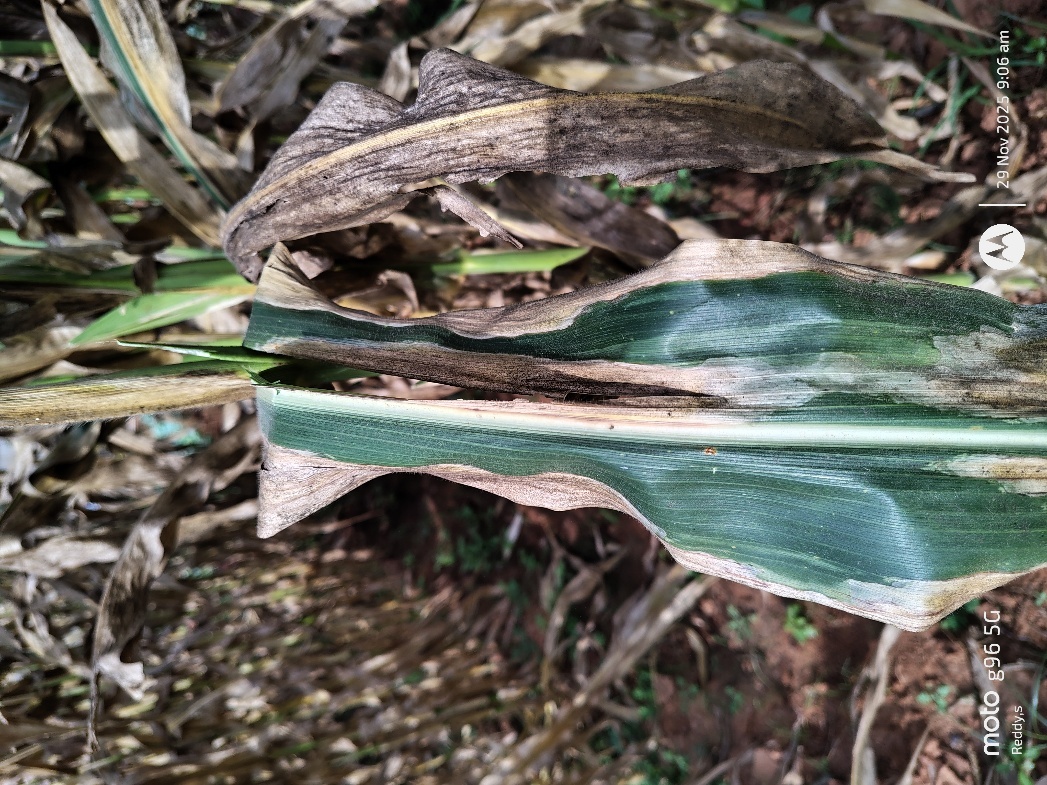 |
